# Effects of Cataract Surgery on Vision-Related Quality of Life in Patients with Retinitis Pigmentosa and the Predictive Factors of Quality of Life Improvement

**DOI:** 10.1155/2021/3846867

**Published:** 2021-09-13

**Authors:** Gen Miura, Takayuki Baba, Tomoaki Tatsumi, Hirotaka Yokouchi, Shuichi Yamamoto

**Affiliations:** Department of Ophthalmology and Visual Science, Chiba University Graduate School of Medicine, Chiba, Japan

## Abstract

**Purpose:**

To determine the effects of cataract surgery and preoperative factors on the vision-related quality of life (QOL) in patients with retinitis pigmentosa (RP).

**Materials and Methods:**

This was a prospective, interventional study of 54 patients diagnosed with RP. The 25-item National Eye Institute Visual Function Questionnaire (NEI VFQ-25) was used to determine the QOL before and after the cataract surgery. The correlations between the scores of the questionnaire and the best-corrected visual acuity (BCVA), macular structure, and degree of improvement of the NEI VFQ-25 scores were also determined.

**Results:**

Statistically significant improvements were observed in the BCVA and all of the NEI VFQ-25 subscale scores except for color vision. The improvement of general vision was the largest. The postoperative BCVA of the better-seeing eye was more strongly and significantly correlated with the postoperative NEI VFQ-25 scores than that of the worse-seeing eye. All of the postoperative NEI VFQ-25 scores were significantly correlated with the length of the ellipsoid zone (EZ) of the photoreceptors. No significant correlation was found between the preoperative general vision, near vision, mental health scores, and EZ length. All of the preoperative NEI VFQ-25 scores except the social function and mental health scores were negatively and significantly correlated with the degree of improvement of the NEI VFQ-25 score. The EZ length was significantly correlated with the degree of improvement of the NEI VFQ-25 scores of the general vision, distance vision, mental health, dependency, and composite 9 scores.

**Conclusions:**

Cataract surgery can significantly improve the NEI VFQ-25 scores in RP patients. The EZ length can be used to predict the postoperative VFQ scores. We conclude that the NEI VFQ-25 is a useful method to evaluate the impact of cataract surgery on the BCVA in patients with RP.

## 1. Introduction

Retinitis pigmentosa (RP) is an inherited retinal disease associated with a progressive degeneration of the photoreceptors and subsequent irreversible reduction of visual function. The prevalence of RP is about 1 in 4000 worldwide, and cataracts are a major complication in RP patients of any hereditary form [1, 2]. It has been reported that the age of onset of cataracts in RP patients is younger than that of normal individuals [3]. Importantly, the cataracts can have a negative impact on the quality of life (QOL) of RP patients.

Earlier studies have shown that cataract surgery in RP patients improved the postoperative mean visual acuity [2] and that the findings obtained with the Humphrey visual field analyzer and optical coherence tomography (OCT) could be used to predict the visual outcome of cataract surgery in patients with RP [4]. Other research studies on the visual functions of RP individuals before and after cataract surgery have been published, but a search of Medline/PubMed did not extract any publications reporting on the impact of cataract surgery on the vision-related quality of life (QOL) in patients with RP. Previous reports showed that cataract surgery led to improvements in the visual acuity and in real-life activities and emotional and social life components [5]. Despite the conclusions that cataract surgery is important for RP patients' QOL, it is currently unclear what the impact of cataract surgery has on the vision-related QOL in RP patients.

The 25-item National Eye Institute Visual Function Questionnaire (NEI VFQ-25) is used to evaluate the vision-related factors that are associated with the individual's QOL. The NEI VFQ-25 is made up of 25 questions that addressed 12 aspects of daily living: the general health, general vision, near vision, distance vision, driving, peripheral vision, color vision, ocular pain, role limitation, dependency, social function, and mental health [6]. By evaluating the NEI VFQ-25 before and after cataract surgery, it should be possible to determine what the impact of the cataract has had on the QOL of the patients. In addition, it should be possible to determine the preoperative factors that affected the postoperative QOL. It is often difficult to predict the postoperative visual function after lensectomy because of the effects of RP alone on the visual function.

Thus, the purpose of this study was to determine the degree of change of the NEI VFQ-25 scores after cataract surgery in RP patients. One other purpose was to determine the preoperative factors that were significantly associated with postoperative improvements of the NEI VFQ-25 scores.

## 2. Materials and Methods

This was a prospective, interventional study of 80 eyes of 54 patients clinically diagnosed with RP who underwent cataract surgery from January 2009 to January 2018 at the Chiba University Hospital.

The diagnosis of RP was made by a progressive increase in night blindness, constriction of the visual field, photophobia, reduced or abolished waveform of electroretinograms (ERG), and ophthalmoscopic findings, viz., bone-spicule-like pigment clumping, attenuated retinal vessels, and optic disc pallor. Only bilateral typical RP patients were studied. Patients with central RP, sectorial RP, retinitis punctata albescens, and RP sine pigmento were excluded. Patients with diabetic retinopathy, uveitis, macular lesions such as vitreous macular traction syndrome, macular edema, epiretinal membrane, macular hole, and refractive errors (spherical equivalents) greater than ±6 diopters were also excluded.

The protocol of this study was approved by the Institutional Review Board of the Chiba University Graduate School of Medicine, and the study adhered to the tenets of the Declaration of Helsinki. All patients were informed about the purpose of this study and the procedures used, and informed consent was obtained from all of them.

The best-corrected visual acuity (BCVA) and NEI VFQ-25 scores were evaluated before and 3 months after the cataract surgery. Hereditary type, ellipsoid zone (EZ) length, and central foveal thickness (CFT) were evaluated before the cataract surgery.

The BCVA was measured monocularly using a Japanese standard Landolt ring chart (System Charts SC-2000 Nidek Instruments, Gamagori, Japan) at 5 m, and the decimal visual acuities were converted to the logarithm of the minimum angle of resolution (logMAR) units for the statistical analyses. The BCVA was investigated for the better-seeing eye and the worse-seeing eye.

EZ length and CFT are known to be parameters that correlate with visual function and RP progression [7, 8]. We investigated whether postoperative vision-related QOL could be estimated by evaluating the correlation between these parameters and postoperative NEI VFQ-25. The length of the EZ and CFT were determined by examining the images that were obtained with the spectral-domain optical coherence tomographic (OCT) Spectralis device (Heidelberg Engineering, Heidelberg, Germany). The 9 mm horizontal and vertical scans of 100 averaged images through the fovea were used. Only OCT scans of good quality, i.e., >25 dB, were used for the measurements. The average of the horizontal and vertical EZ lengths and the CFT was used for the statistical analyses. The EZ length and CFT were measured independently by two of the authors (GM and TB) in a masked way. We also classified all patients into 3 groups according to the EZ status at the fovea: Grade 1, EZ not visible; Grade 2, EZ abnormal or discontinuous; and Grade 3, EZ normal [9]. In Gr2 cases, we measured the total EZ length minus the length of disruption of the EZ just beneath the fovea as EZ length.

The vision-related QOL was measured with NEI VFQ-25 preoperatively and at 3 months after the cataract surgery. Patients answered the NEI VFQ-25 Japanese version [10] to evaluate the subjective symptoms and quality of life. The NEI VFQ-25 is made up of 25 questions that addressed 12 items of daily living: general health, general vision, near vision, distance vision, driving, peripheral vision, color vision, ocular pain, role limitation, dependency, social function, and mental health. In this study, nine subscales (general vision, near vision, distance vision, peripheral vision, color vision, role limitation, dependency, social function, and mental health) are used and the compo 9 which comprehensively evaluates these nine subscales.

We also determined the relationship between the preoperative and postoperative BCVA, EZ length, CFT, and degree of improvement of the NEI VFQ-25 scores. The significance of the differences in the values before and after surgery was determined by Mann–Whitney tests. Spearman's rank tests were used to determine the significance of any correlation. A *P* value < 0.05 was taken to be statistically significant.

## 3. Results

All eyes underwent phacoemulsification and implantation of a yellow-colored acrylic foldable intraocular lens through a superior sclera-corneal incision without any complications. The cataracts in all cases were nuclear, cortical, subcapsular, or a combination of them and either Grade 2 or Grade 3 on the Emery-Little classification [11]. There were 26 men and 28 women whose mean age at the baseline was 62.3 ± 11.2 years. Three patients had autosomal dominant, 10 patients had autosomal recessive, and 38 had sporadic RP. The type of inheritance of the remaining 3 was not known.

The preoperative EZ was classified as Grade 1 (EZ not visible) in 5 patients, Grade 2 (EZ abnormal or discontinuous) in 18 patients, and Grade 3 (EZ normal) in 31 patients. The mean preoperative EZ length was 2721.5 ± 23.6 *μ*m, and the mean CFT was 213.7 ± 69.6 *μ*m. The mean compo 9 scores of Grades 1, 2, and 3 before and after surgery were 29.2 and 40.1 (*P* = 0.10), 48.8 and 56.1 (*P* = 0.002), and 54.4 and 65.8 (*P* < 0.0001), respectively.

Twenty-six patients had binocular surgery and 28 patients had monocular surgery. There were no cases in which the BCVA decreased to less than the preoperative BCVA. There were no cases of posterior capsular opacification that required neodymium : YAG laser capsulotomy, intraocular lens dislocation, or macular edema within the observation period of 3 months after the surgery.

The BCVA of the better- and worse-seeing eyes and the NEI VFQ-25 scores before and after the cataract surgery are shown in Table 1. There were significant improvements in the BCVA and all NEI VFQ-25 subscale score except color vision, and the improvement was the largest for the general vision score.

The correlations between the postoperative BCVA in the better and worse eyes and postoperative NEI VFQ-25 score are shown in Table 2. The postoperative BCVA of the better-seeing eye was significantly correlated with all postoperative NEI VFQ-25 subscale scores. In addition, a stronger correlation coefficient was found between the NEI VFQ-25 score and the BCVA of the better-seeing eye than with the worse-seeing eye.

The correlations between the EZ length, CFT, and NEI VFQ-25 score before and after the cataract surgery are shown in Table 3. All postoperative NEI VFQ-25 scores were significantly correlated with the EZ length. Similarly, the CFT was significantly correlated with the postoperative NEI VFQ-25 scores except for color vision, but the correlation coefficient tended to be lower than that of the EZ length. On the other hand, no correlation was found between the preoperative general vision, near vision, mental health scores, and EZ length.

The correlations between the preoperative NEI VFQ-25 score and the degree of improvement of the NEI VFQ-25 score and between the preoperative NEI VFQ-25 score and EZ length are shown in Table 4. The preoperative NEI VFQ-25 scores except social function and mental health were significantly correlated with the improvement of the NEI VFQ-25 score. The EZ length and degree of NEI VFQ-25 score improvement of general vision, distance vision, mental health, dependency, and composite 9 score were significantly correlated. Both the preoperative BCVA and CFT were not significantly correlated with the degree of improvement of any NEI VFQ-25 subscale scores.

## 4. Discussion

Several earlier studies on the vision-related QOL of cataract patients reported significant improvements in the vision-related QOL, and the patients had reduced depressive symptoms after the cataract surgery [12–14]. In our RP patients, the postoperative NEI VFQ-25 scores except color vision were also significantly improved compared to the preoperative scores (Table 1). However, our results had less improvement than that reported earlier in cataract cases without other ocular diseases [15]. This difference is probably due to the retinal degeneration related to the RP.

The BCVA of both the better-seeing eye and worse-seeing eye were significantly correlated with the postoperative NEI-VFQ scores. However, the BCVA of the better-seeing eye tended to have a higher coefficient of correlation that excluded the effects of lens opacification after the cataract surgery (Table 2). Earlier studies reported that the vision-related QOL decreased significantly when there was a severe visual impairment in one eye but there was no impairment in the fellow eye [16]. Earlier studies targeting unilateral AMD [17] and BRVO [18] showed that the vision-related QOL was significantly reduced compared to a control group, and the VFQ-25 scores were correlated with the BCVA of the eye involved even with good BCVA of the fellow eye. As stated above, it has been reported that BCVA of the worse-seeing eye is more related to the VFQ score in studies targeting unilateral diseases. However, the results of this study which targeted RP patients with visual impairments in both eyes suggested that the visual function of the better-seeing eye may be more related to the NEI-VFQ scores in RP patients.

We also performed an intergroup analysis of monocular surgery (*n* = 28) and binocular surgery (*n* = 26). Significant improvement of the VFQ score except for color vision in both groups was seen, and all VFQ score improvements were not statistically significantly different between two groups (data not shown). Therefore, these results indicated that there were no big differences in VFQ score improvement between monocular and binocular surgery groups.

Our results showed that the EZ length and NEI VFQ-25 scores were significantly correlated before and after the surgery. Because the coefficient of correlation of the preoperative group tended to be lower than that of the postoperative group, this suggested that the lens opacity affected the correlation between the outer retinal structure and the NEI-VFQ scores.

The significant correlations between the degree of postoperative improvements in the NEI-VFQ-25 subscale scores and preoperative explanatory variables are shown in Table 4. Significant correlations were found between the EZ length and the degree of improvement in general vision, distance vision, mental health, dependency, and composite 9 score. Thus, a significant improvement of the VFQ may be expected after cataract surgery especially in those subscale scores.

The mean compo 9 score improved in all OCT grades after surgery, but showed a statistically significant improvement only in the OCT Grade 2 and 3 groups. The mean compo 9 score of both before and after surgery decreased as the OCT grade decreased. These results indicated that the preoperative outer retinal structure measured by OCT that reflect macular function could be important parameters to predict the pre- and postoperative VFQ score. It has been reported that foveal EZ morphology is an important parameter for predicting postoperative visual results [4] and that visual acuity and VFQ are correlated [16]. Their results are consistent with our conclusion about correlation between EZ length and VFQ score. On the other hand, the correlation between EZ length and the degree of improvement in social function and role limitation was not significant. These findings suggested that the improvement in those subscales may be related to factors other than the disease stage and anatomical structure of the retina.

Significant negative correlations were found between the preoperative VFQ scores and the degree of improvement in all subscale scores except the social function and mental health scores. These results indicate that even if the preoperative VFQ score is low, cataract surgery can be expected to improve the VFQ score. There was no significant correlation between the social function, mental health scores, and EZ length which indicates that different factors may be associated with their improvement. The EZ length may be a predictor of the degree of improvement in some VFQ subscale scores, but both the preoperative BCVA and CFT were not.

There are several limitations in this study. The first was the small number of patients which limited the strength of the statistical analyses. Second, the short follow-up period of 3 months may be the reason for the low rate of postoperative complications such as posterior capsule opacification, intraocular lens dislocation, or progression of retinal degeneration. Therefore, the conclusions drawn from this study regarding the long-term impact of cataract surgery must be carefully interpreted. Another limitation was that both binocular and monocular surgery cases were included in the analyses. In addition, there was a bias in the number of patients of different preoperative OCT grades which could have affected the changes in the VFQ scores. Third is the lack of evaluation of depressive state. This is important because visual impairments are major risk factors for depressive symptoms in elderly people [19]. Previous study showed that patients with improved QOL due to cataract surgery improved cognitive function and depression [20]. Sainohira et al. reported that visual function impairments and vision-related QOL are associated with a depressive state, and the general health condition is related to anxiety in RP patients [21]. However, we have not evaluated depressive state related to cataract surgery in this study. Finally, we have not performed a genetic testing, so the differences in VFQ scores before and after surgery for each type of RP were not determined.

## 5. Conclusions

The postoperative VFQ score was significantly improved compared to the preoperative score after cataract surgery even if the preoperative VFQ score is low. The EZ length was significantly correlated with the VFQ score especially after cataract surgery, and the visual function of the better-seeing eye may be more related to vision-related QOL in RP patients. Significant correlations were found between the EZ length and the degree of improvement in part of the VFQ score. We conclude that the EZ length may be an important predictor of the amount of improvement in the VFQ subscale scores. NEI VFQ-25 is a helpful method that can adequately evaluate the impact of cataract surgery on the QOL in patients with RP.

## Figures and Tables

**Table 1:** BCVA and NEI VFQ-25 scores before and after surgery.

	Preoperative	Postoperative	*P* value
BCVA (logMAR units)			
Better	0.371 ± 0.30	0.198 ± 0.30	<0.0001
Worse	0.773 ± 0.57	0.481 ± 0.64	<0.0001
General vision	44.44 ± 18.1	64.40 ± 20.4	<0.0001
Near vision	44.60 ± 25.2	56.17 ± 22.4	<0.0001
Distance vision	49.07 ± 20.1	58.64 ± 19.9	<0.0001
Social function	58.10 ± 26.1	62.96 ± 27.4	0.0304
Mental health	42.60 ± 25.9	55.21 ± 30.2	<0.0001
Role limitation	57.41 ± 24.3	66.44 ± 26.7	<0.0001
Dependency	54.63 ± 29.7	66.36 ± 29.5	<0.0001
Color vision	72.22 ± 26.9	75.46 ± 26.8	0.2352
Peripheral vision	28.70 ± 23.5	36.11 ± 20.4	0.0071
Compo 9	50.99 ± 19.8	60.81 ± 20.7	<0.0001

BCVA: best-corrected visual acuity; better: better-seeing eye; worse: worse-seeing eye; compo 9: composite score of 9 subscales.

**Table 2:** Correlations between postoperative BCVA and postoperative NEI VFQ-25 scores.

Postoperative VFQ-25	Postoperative BCVA			
	Better	*P* value	Worse	*P* value
General vision	-0.471	0.0003	-0.238	0.0835
Near vision	-0.674	<0.0001	-0.316	0.0194
Distance vision	-0.627	<0.0001	-0.384	0.0038
Social function	-0.618	<0.0001	-0.461	0.0004
Mental health	-0.558	<0.0001	-0.486	0.0001
Role limitation	-0.528	<0.0001	-0.328	0.0149
Dependency	-0.652	<0.0001	-0.518	<0.0001
Color vision	-0.543	<0.0001	-0.218	0.1140
Peripheral vision	-0.420	<0.0001	-0.333	0.0134
Compo 9	-0.661	<0.0001	-0.535	<0.0001

VFQ: Visual Function Questionnaire; better: better-seeing eye; worse: worse-seeing eye; compo 9: composite score of 9 subscales. Data are the correlation coefficient.

**Table 3:** Correlations between preoperative EZ length, preoperative CFT, and pre- and postoperative NEI VFQ-25 scores.

	EZ length		CFT	
	Preoperative	Postoperative	Preoperative	Postoperative
General vision	-0.098	0.370^†^	0.116	0.369^†^
Near vision	0.216	0.463^†^	0.337^‡^	0.346^‡^
Distance vision	0.333^‡^	0.541^∗^	0.337^‡^	0.427^†^
Social function	0.348^†^	0.438^†^	0.256	0.377^†^
Mental health	0.217	0.482^†^	0.275^‡^	0.374^†^
Role limitation	0.403^†^	0.426^†^	0.324^‡^	0.300^‡^
Dependency	0.389^†^	0.581^∗^	0.422^‡^	0.488^†^
Color vision	0.361^†^	0.359^†^	0.147	0.255
Peripheral vision	0.315^‡^	0.437^†^	0.184	0.342^‡^
Compo 9	0.336^‡^	0.528^∗^	0.335^‡^	0.431^†^

^∗^*P* < 0.001, ^†^*P* < 0.01, and ^‡^*P* < 0.05. EZ: ellipsoid zone; CFT: central foveal thickness.

**Table 4:** Correlations between postoperative improvements in NEI VFQ-25 subscales and preoperative explanatory variables.

Postoperative improvement	Preoperative factors			
	VFQ-25	EZ length	BCVA	CFT
			Better/worse	
General vision	-0.482^†^	0.431^†^	-0.063/0.033	0.252
Near vision	-0.526^∗^	0.251	0.093/0.229	-0.039
Distance vision	-0.373^†^	0.282^‡^	-0.044/0.204	0.119
Social function	-0.242	0.175	-0.055/0.123	0.220
Mental health	-0.143	0.454^†^	-0.094/-0.089	0.213
Role limitation	-0.435^†^	0.079	0.095/0.193	0.006
Dependency	-0.336^‡^	0.289^‡^	-0.033/-0.086	0.095
Color vision	-0.354^†^	-0.005	0.110/0.192	0.153
Peripheral vision	-0.526^∗^	0.091	0.163/0.040	0.160
Compo 9	-0.235	0.350^†^	0.028/0.162	0.199

^∗^*P* < 0.001, ^†^*P* < 0.01, and ^‡^*P* < 0.05. VFQ: Visual Function Questionnaire; EZ: ellipsoid zone; BCVA: best-corrected visual acuity; better: better-seeing eye; worse: worse-seeing eye; CFT: central foveal thickness.

## Data Availability

All the data supporting our findings are contained within the manuscript.
